# Supplementary material for: Spontaneous formation of nanoparticles on electrospun nanofibres
Source: Nat Commun. 2018 Nov 9;9:4740. doi: 10.1038/s41467-018-07243-5 (PMC6226441; doi:10.1038/s41467-018-07243-5)
Supplement: Supplementary file 1 — Supplementary Information [file 41467_2018_7243_MOESM1_ESM.pdf]

Supplementary information

Spontaneous formation of nanoparticles on electrospun nanofibres

N. Radacsi et al.

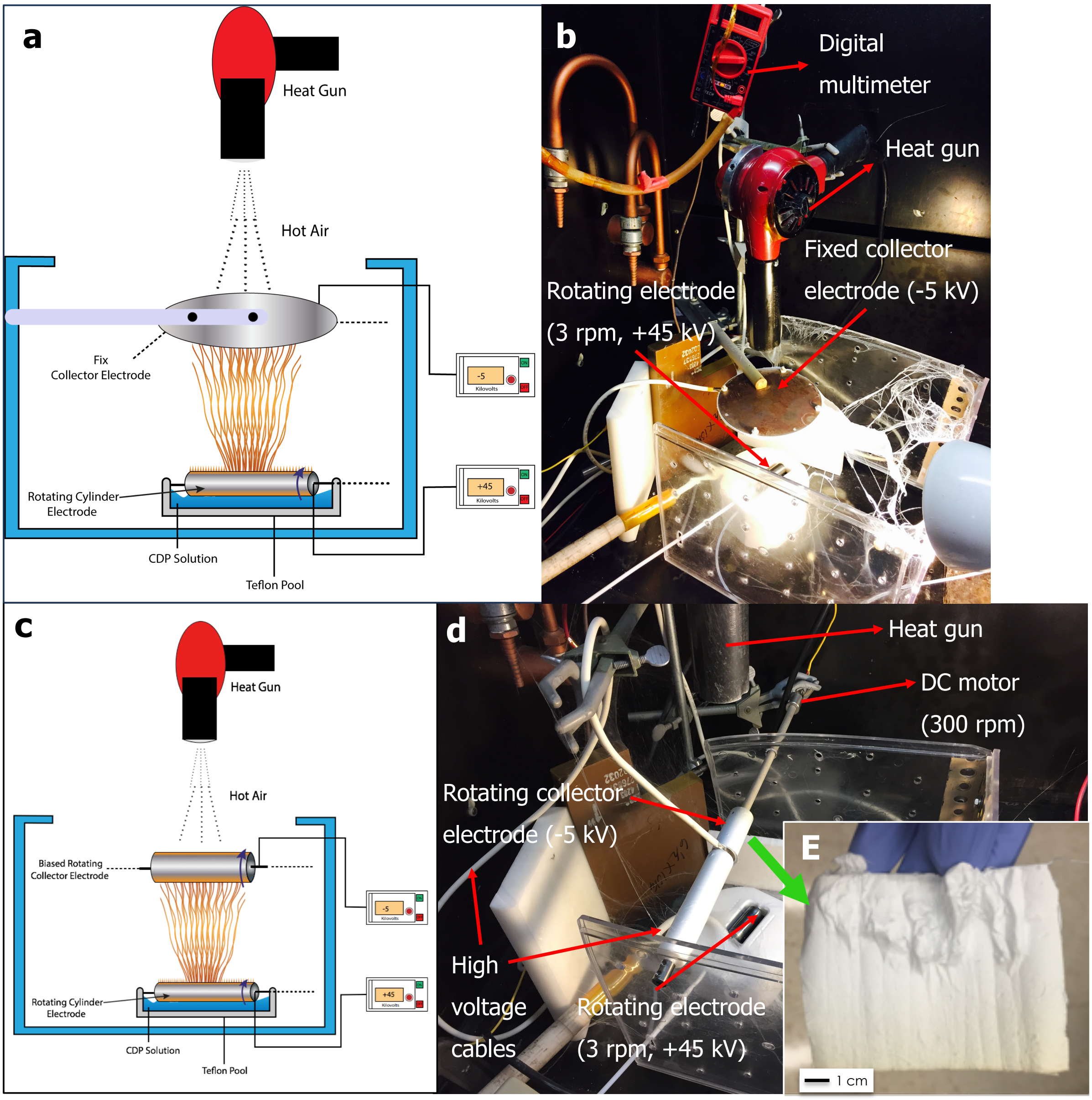

Supplementary Figure 1. Illustrations and pictures of the used experimental setup configurations. **a** Schematic illustration of the nozzle-free electrospinning setup with the fixed collector electrode. **b** Photograph of the experimental setup with the fixed collector electrode, showing the digital multimeter that is connected in series with the negative DC bias power supply and the heat gun. **c** Schematic illustration of the nozzle-free electrospinning setup with the rotating collector electrode. **d** Photograph of the experimental setup with the rotating collector electrode. **e** Picture of the collected electrospun CDP-PVP-PANI sample after removal from the rotating collector electrode.

CDP concentration [mg mL^{-1}]	PVP concentration [mg mL^{-1}]	PVA concentration [mg mL^{-1}]
10	12	15
25	15	30
30	20	
35	25	
40	27	
50	29	
60	30	
70	33	
120	35	
	45	
	60	
	80	

Supplementary Table 1. The different investigated concentrations of CDP/PVP/PVA. The values in the columns are independent from each other.

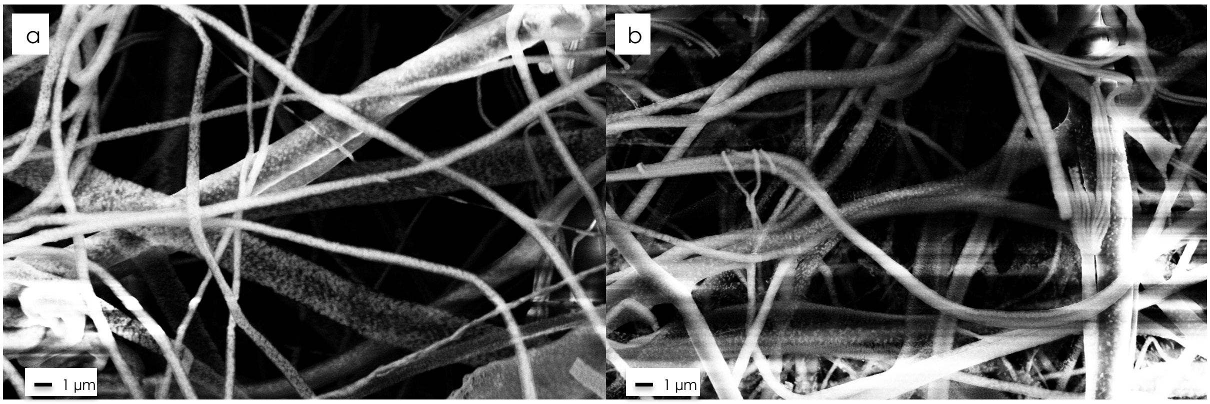

Supplementary Figure 2. SEM images of samples using 30 mg mL^{-1} CDP and 30 mg mL^{-1} PVA concentrations, showing nanoparticles on the fibre surfaces. **a** Sample prepared from solution with 0.1 mg mL^{-1} PANI concentration **b** Sample prepared from solution without addition of PANI. Scale bar is 1 µm for both images.

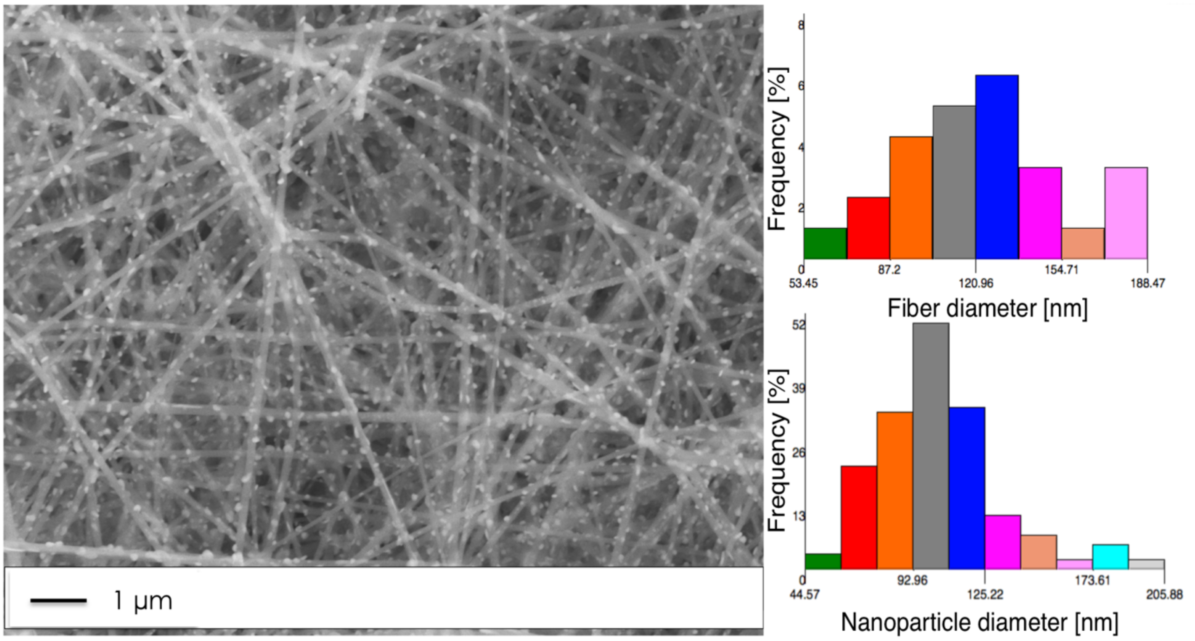

Supplementary Figure 3. Using 50 mg mL^{-1} CDP 30 mg mL^{-1} PVP and 0.1 mg mL^{-1} PANI concentration in the solution, a nanoparticle decorated nanofiber composite mat with a mean diameter of the nanofibers being 123.9 ± 32 nm, and the mean diameter of the nanoparticles being 104.5 ± 28 nm was achieved. The nanoparticle density was 29 particles µm^{-2}.

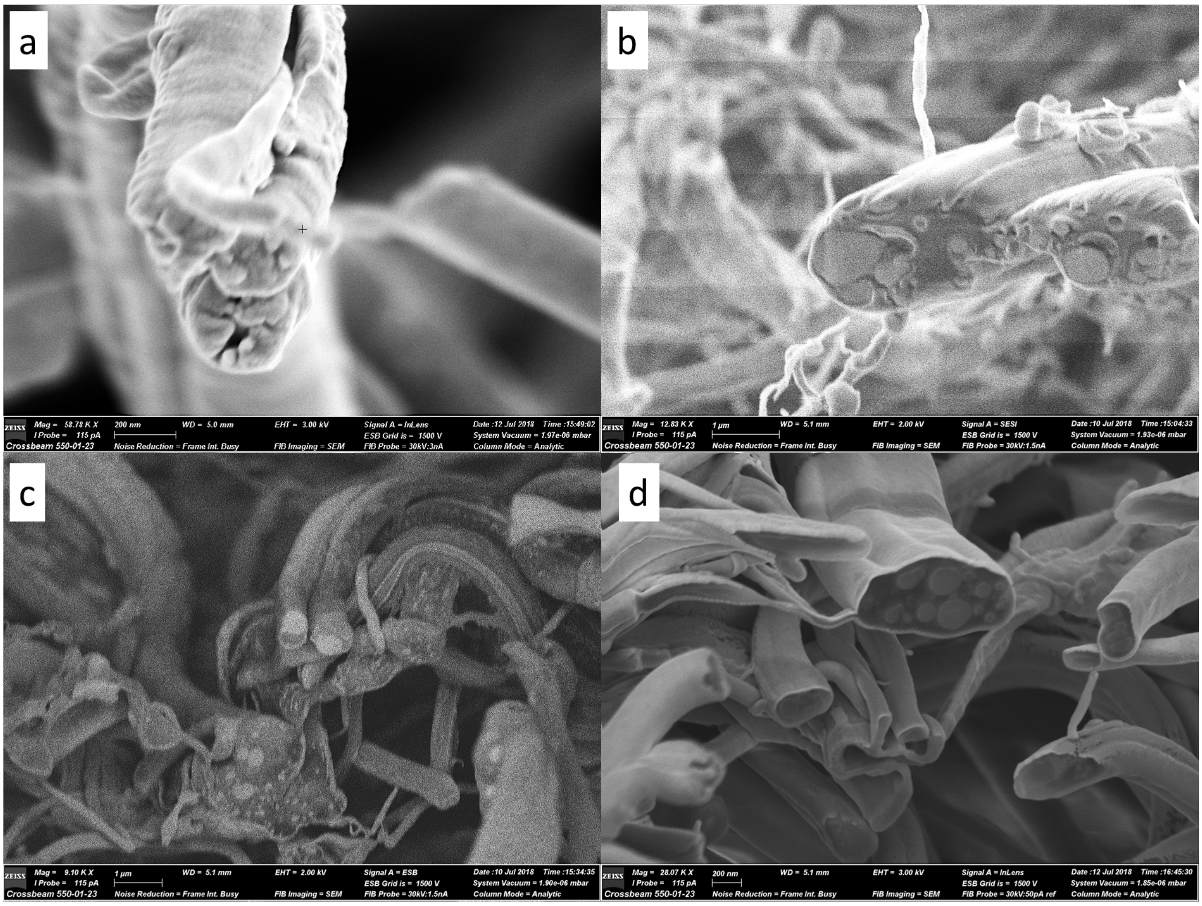

Supplementary Figure 4. Cross-section of electrospun nanofibers of CDP-PVP-PANI, showing CDP nanoparticles inside the fibres. The fibres were cut by focused ion beam (FIB). **a** The scale bar is 200 nm. **b** The scale bar is 1 μm. **c** The scale bar is 1 μm. **d** The scale bar is 200 nm.

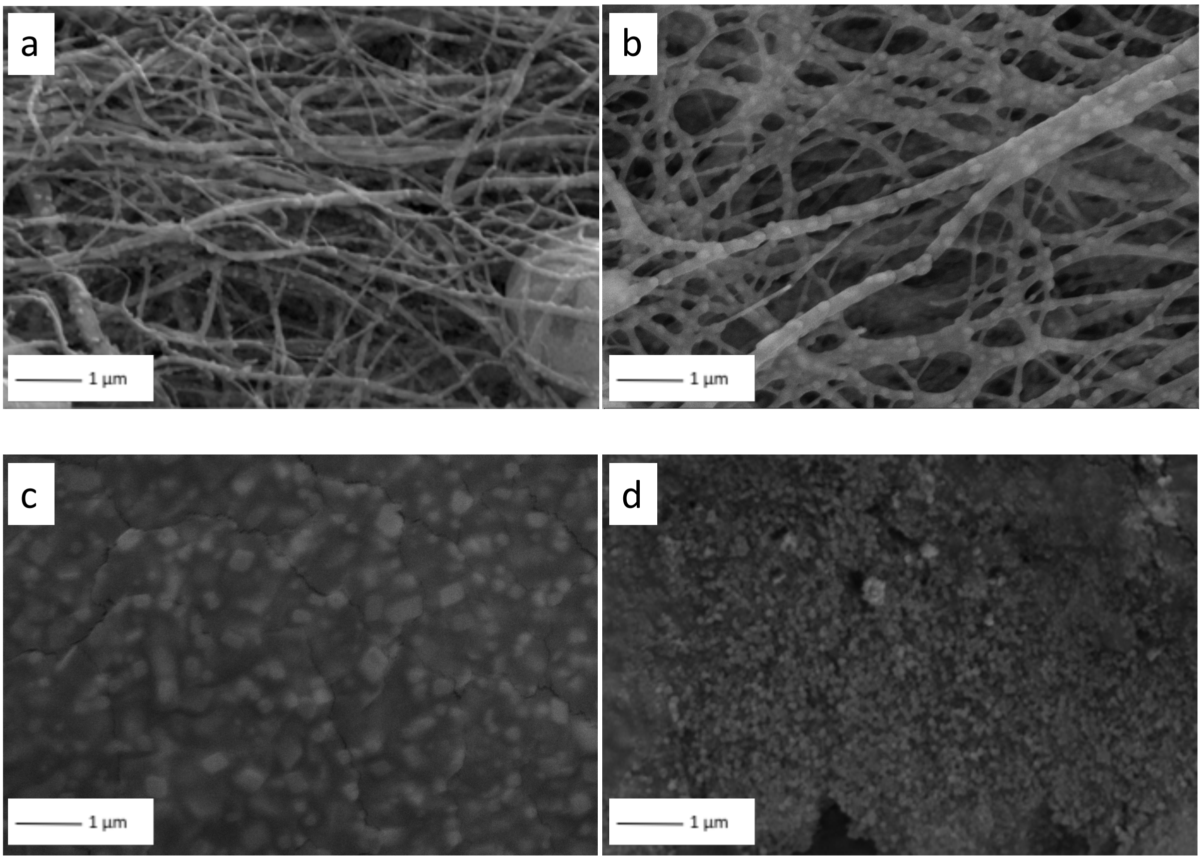

Supplementary Figure 5. Electrospun CDP-PVP-PANI sample investigated under different humidity conditions for 7 days. **a** The as-spun nanoparticle decorated nanofibers imaged right after electrospinning. **b** The sample stored for 7 days under 0% RH (vacuum) **c** Sample stored at 55% RH. **d** Sample stored at 73% RH.

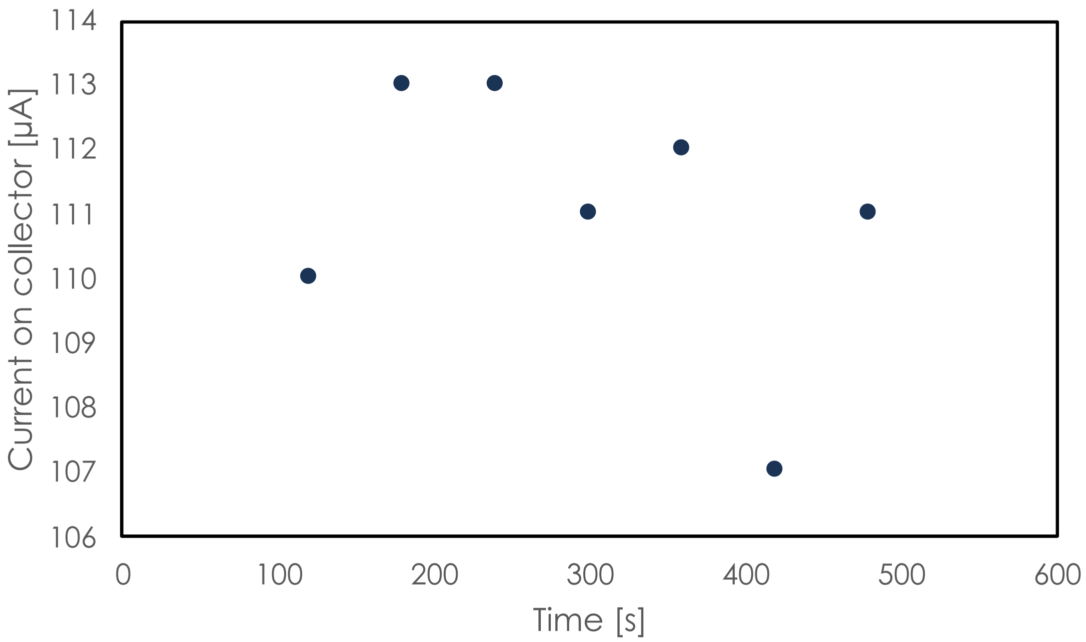

Supplementary Figure 6. Measured current over time on the collector plate.

Supplementary Note 1.

Furnace treatment was applied for the reduction of the polymer a. The electrospun nanofibrous sample mat (310 mg) was placed in a Barnstead box furnace (Supplementary Figure 7). After heating the sample to 200 °C with 5 °C min^{-1}, and holding the temperature for 1 hour, the temperature was ramped to 230 °C with 5 °C min^{-1}, where it was again held for 1 hour. Then the temperature was increased to 300 °C with 5 °C min^{-1}. The sample was left at 300 °C for 12 hours in air. The composite lost 38.5% of the PVP content (as the weight decreased from 310 mg to 191 mg). During the process, the sample changed colour from white to brown. Then the treated sample was placed on top of a Toray carbon paper, above 100 mg Platinum(II) acetylacetonate powder. After an MOCVD process, where the sample was heated to 210 °C in a vacuum oven (-27 inch.Hg pressure) in the presence of the platinum precursor and 2 mL deionized water, 62 mg Pt was deposited on the nanofibrous CDP mat. The platinum particles diffused into the sample and deposited homogeneously, increasing the sample mass to 253 mg. The sample was then sieved over a 53 µm sieve, and hand-spread on a Toray carbon paper. Finally, the powder on the carbon paper was pressed with the 2” diameter anode+electrolyte half-cell (prepared by SAFCell standard procedure) with 1 kton pressure for 3 seconds.

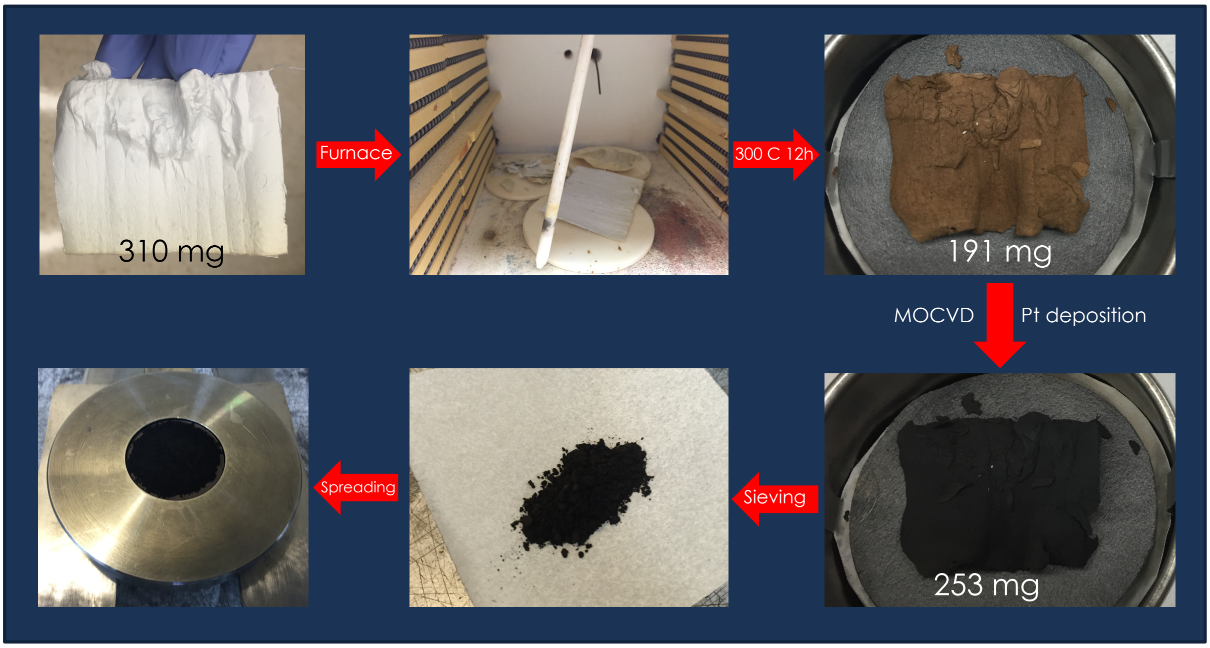

Supplementary Figure 7. The electrospun sample mat (310 mg) was placed in a box furnace (300 °C heat treatment for 12 hours in air). Upon removing the 38.5% of PVP, the weight decreased to 191 mg. The sample changed colour from white to brown. Then Pt was deposited on the sample by MOCVD process. The sample was then sieved over a 2 µm sieve, and hand-spread on a Toray carbon paper, which finalised the cathode assembly.

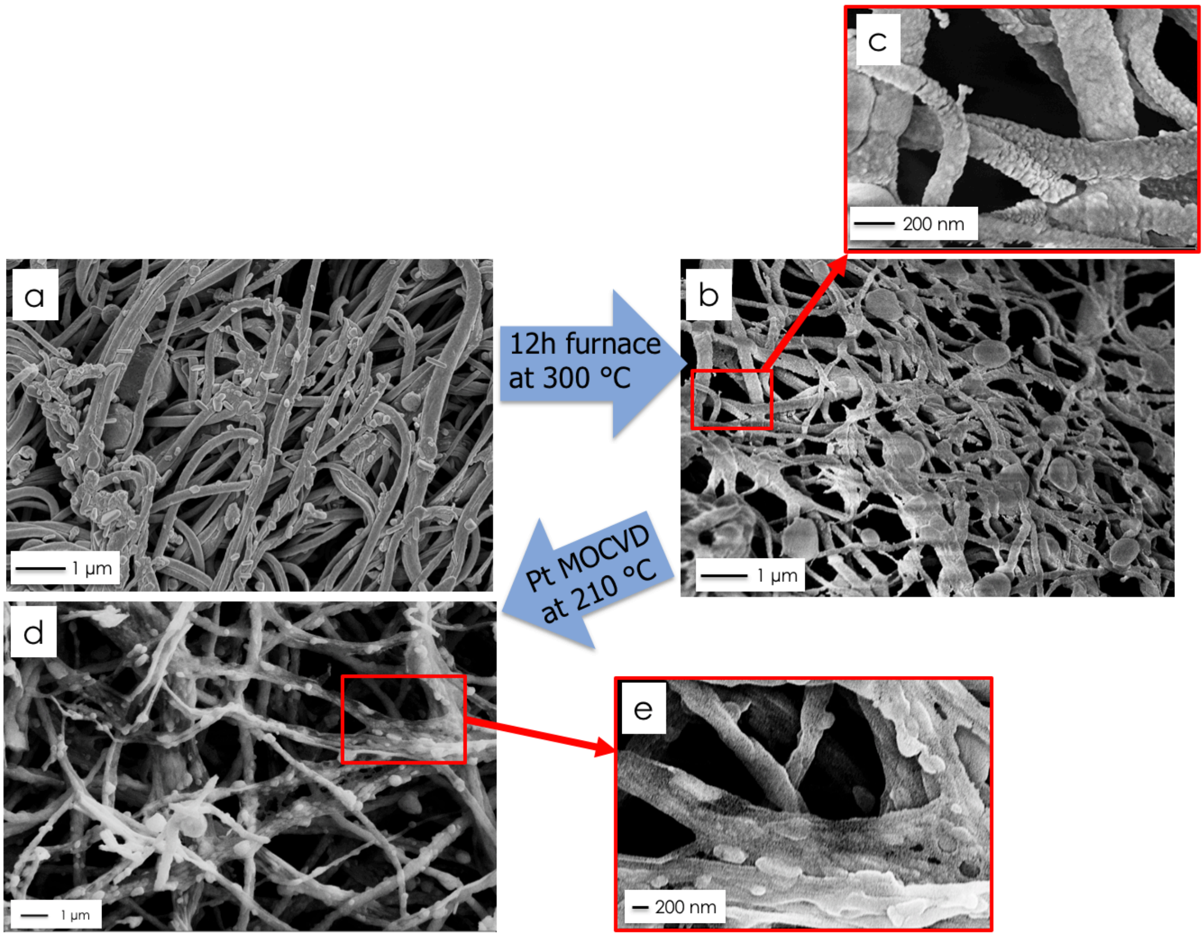

Supplementary Figure 8. SEM images of the electrospun CDP-PVP-PANI sample before furnace treatment, after the 12-hour furnace treatment in air, and furnace treatment plus additional 15-hour MOCVD in vacuum. **a** The 'as-spun' sample, before the 12-hour furnace treatment in air. **b** Sample after the 12-hour furnace treatment in air. **c** Magnified image of the sample after the 12-hour furnace treatment in air. **d** Sample after the 12-hour furnace treatment in air and additional 15-hour MOCVD in vacuum. **e** Magnified image showing the Pt nanoparticles on the electrospun fibres.

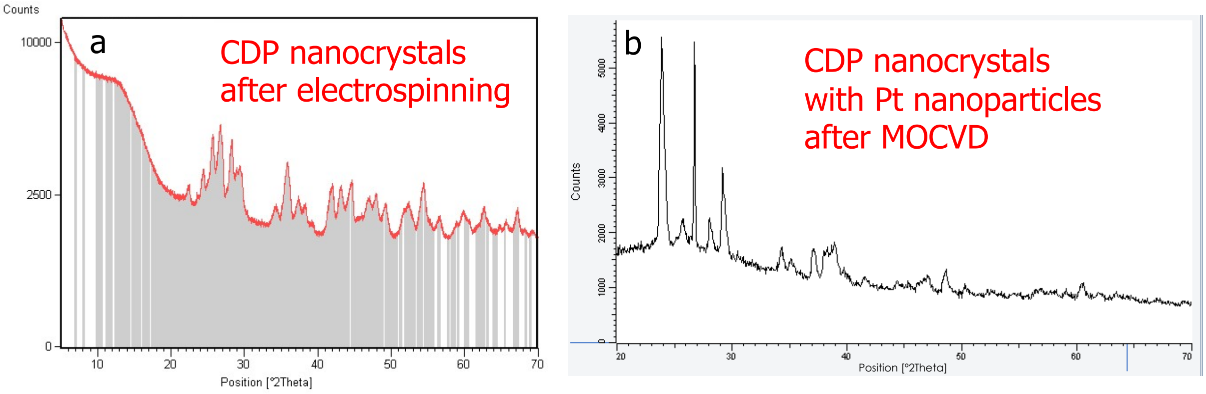

Supplementary Figure 9. X-ray diffraction pattern of the electrospun CDP-PVP-PANI showing the monoclinic form **a** The as-spun sample showing the monoclinic crystalline structure of CDP. **b** The sample after Pt deposition by MOCVD method showing the monoclinic crystalline structure of CDP.

Supplementary Note 2.

The 2” diameter electrode was tested in a symmetric cell mode using H_2 gas on the anode and cathode. Impedance measurement of cell with standard SAFCell Pt on carbon “anode” as pseudo-hydrogen reference electrode, and electrospun electrode on “cathode” as working electrode was performed. High frequency intercept with real axis was taken to measure all electronic, plus electrolyte layer protonic losses across cell, with area specific resistance value of 0.32 Ω cm^2. Real axis difference between high and low frequency intercepts measures the total electrode resistances, with a value certainly less than 70 mΩ cm^2 (0.07 Ω cm^2), for both electrodes. However, it is hard to estimate the exact value for the low frequency intercept with real axis, as the arc is so small, and the data became noisy at lower frequencies (Supplementary Figure 10a). Hence, a voltage versus current curve was taken in same symmetric hydrogen setup. From the data it is clear that the curve is linear in nature, and we can then assume that the cell is in the “linear Butler Vohlmer” region, where the slope of the data corresponds to the total ohmic (electrical + protonic) and non-ohmic (electrodes) losses across cell. Subtracting out the ohmic area specific resistance from this slope, gives a value of 50 mΩ cm^2 (0.05 Ω cm^2), for both electrodes (Supplementary Figure 10b). The value for the standard SAFCell Pt on carbon anode is 30 mΩ-cm2 (0.03 Ω cm^2). This means the value for the electrospun electrode in symmetric hydrogen is around 20 mΩ cm^2 (0.02 Ω cm^2).

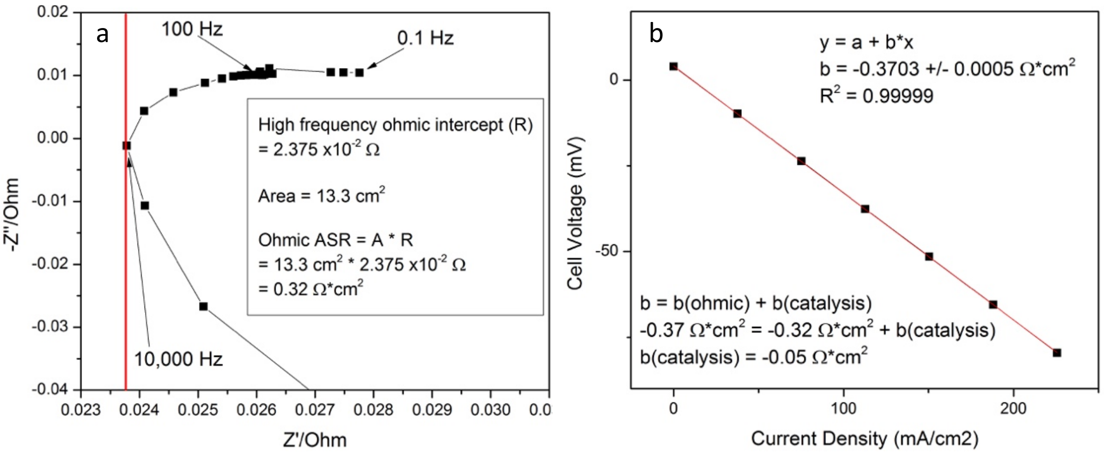

Supplementary Figure 10. Impedance measurements on electrospun cathode assembly taken in a symmetric hydrogen setup. **a** electrochemical impedance of cell with standard SAFCell Pt on carbon “anode” as pseudo-hydrogen reference electrode, and the electrospun electrode on “cathode” as working electrode. **b** Voltage versus current curve. Subtracting out the ohmic area specific resistance from this slope, gives a value of 50 mΩ-cm^2, for both electrodes. The value for the standard SAFCell Pt on carbon anode is 30 mΩ-cm^2. This means the value for the electrospun electrode in symmetric hydrogen is around 20 mΩ-cm^2 (0.02 Ω-cm^2).

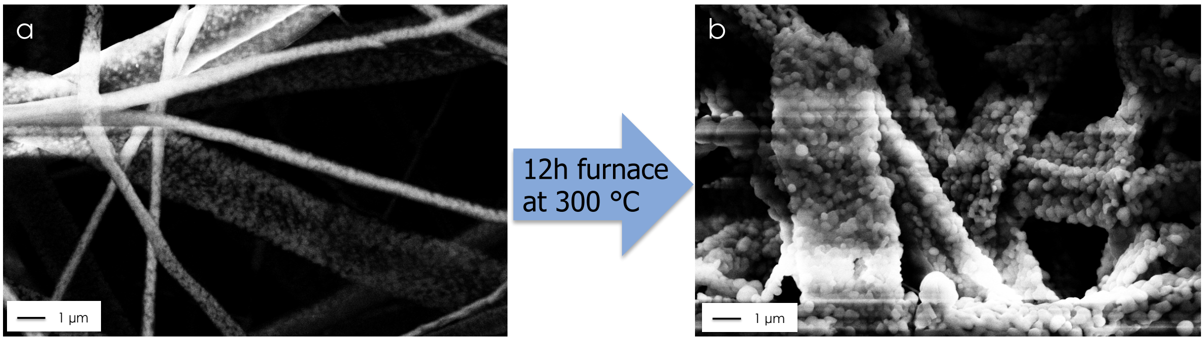

Supplementary Figure 11. SEM images of the electrospun CDP-PVA-PANI sample. **a** The ‘as-spun’ sample, before the 12-hour furnace treatment in air. **b** The sample after the 12-hour furnace in air.

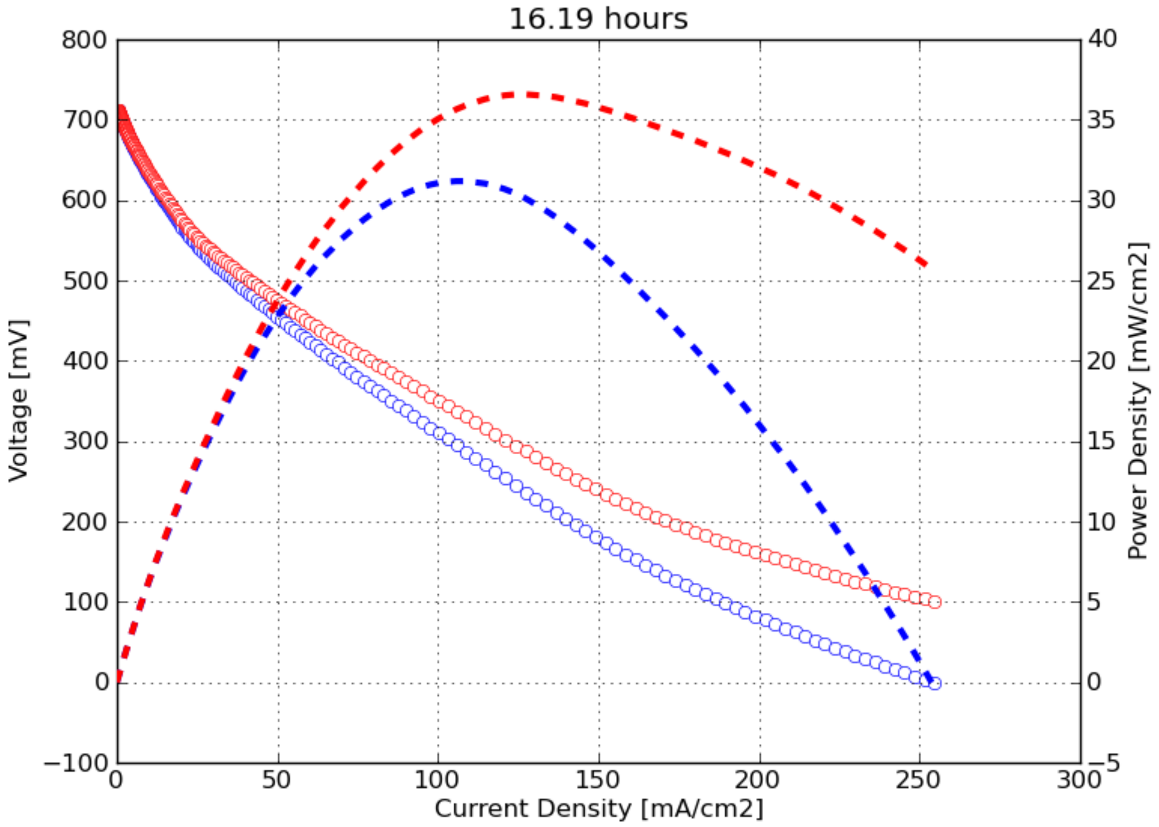

Supplementary Figure 12. Electrochemical performance of the fuel cell made with the electrospun CPD-PVA after 16.19 hours of operation (solution parameters: 30 mg mL^{-1} CDP, 30 mg mL^{-1} PVP and 0.1 mg mL^{-1} PANI concentration). The blue data points show the real I-V curve, whereas the red data is iR-free. The dotted blue and red lines show the power density. Both the cell voltage and power density are well below the performance of the CDP-PVP-PANI samples, or the SAFCell Inc. standard.

Supplementary Note 3.

The temperature and the needleless nature of the process does not seem to be relevant for the CDP nanoparticle formation on the nanofibers. Nanoparticle decorated nanofibers with a needle-based electrospinning apparatus at ambient temperature (Supplementary Figure 13) was fabricated. The electrospun fibres have a diameter of 194.8 ± 12 nm, and the nanoparticles have the average diameter of 69 ± 21 nm.

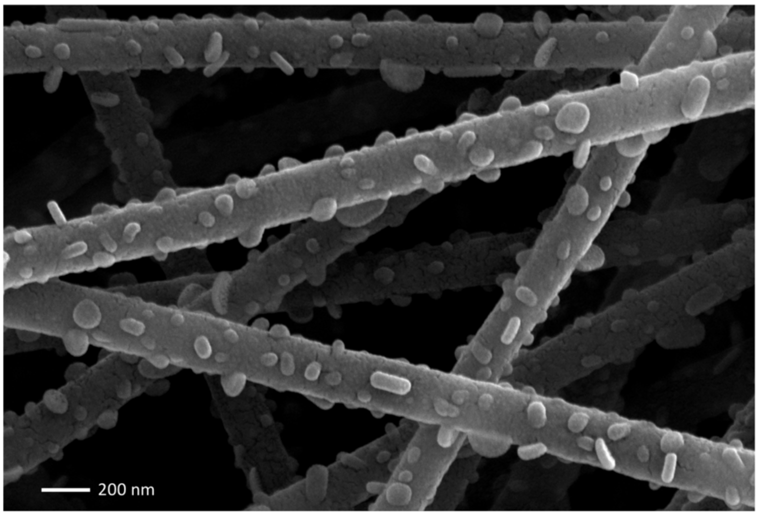

Supplementary Figure 13. Electrospun CDP nanoparticle decorated nanofibers using a conventional, needle-based electrospinning setup. The solution used had 50 mg mL^{-1} CDP concentration, 80 mg mL^{-1} PVP concentration, and 0.1 mg mL^{-1} PANI concentration. Process parameters were the following: 9 kV potential difference and 10 cm distance between the nozzle and the grounded collector, and 0.25 mL solution flow rate.

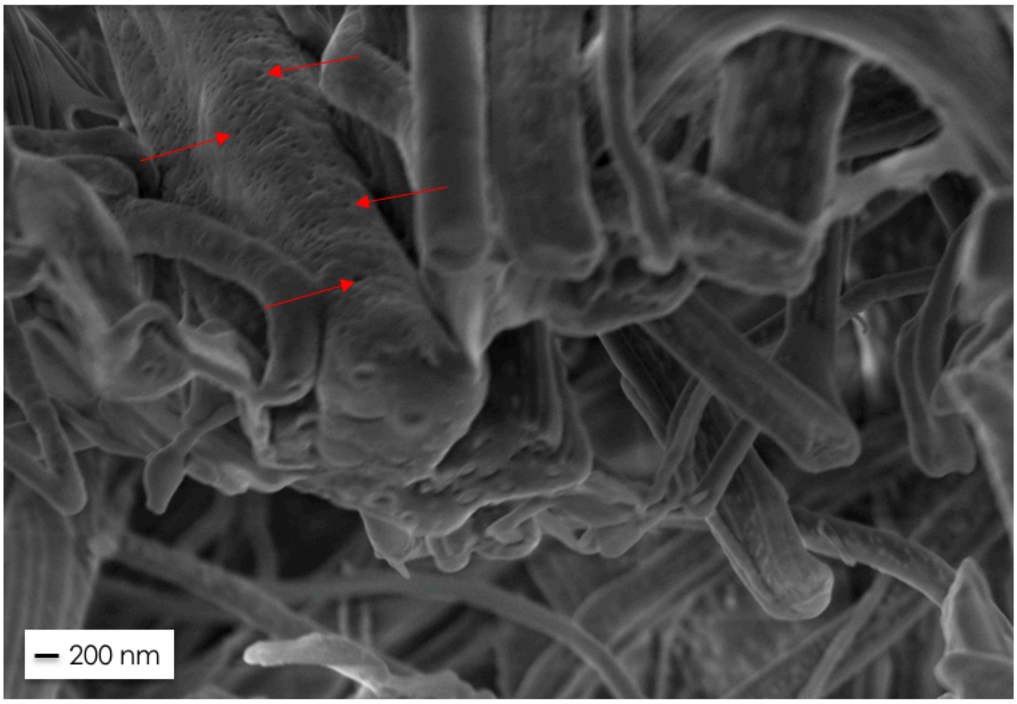

Supplementary Figure 14. SEM images of the electrospun CDP-PVP-PANI sample showing nanopores that can aid the formation of the CDP nanoparticles on the fibre surface. The red arrows point to the pores. The fibres were cut by FIB.
